# Vitamin D strengthens the bladder epithelial barrier by inducing tight junction proteins during *E. coli* urinary tract infection

**DOI:** 10.1007/s00441-019-03162-z

**Published:** 2020-01-13

**Authors:** Soumitra Mohanty, Witchuda Kamolvit, Olof Hertting, Annelie Brauner

**Affiliations:** 1 Department of Microbiology, Tumor and Cell Biology, Division of Clinical Microbiology, Karolinska Institutet and Karolinska University Hospital, SE-17176 Stockholm, Sweden; 2 Department of Women´s and Children´s Health, Division of Pediatric Infectious Diseases, Astrid Lindgren Children’s Hospital, Karolinska Institutet and Karolinska University Hospital, Stockholm, Sweden

**Keywords:** Vitamin D, *Escherichia coli*, Urinary bladder, Occludin, Claudin-14

## Abstract

Tight junction proteins are pivotal to prevent bacterial invasion of the epithelial barrier. We here report that supplementation with vitamin D can strengthen the urinary bladder lining. Vitamin D deficient and sufficient mice were infected with *Escherichia coli (E. coli)* transurethrally to cause urinary tract infection. In addition, bladder biopsies were obtained from postmenopausal women before and after a 3-month period of supplementation with 25-hydroxyvitamin D_3_ (25D_3_) and ex vivo infected with *E. coli*. In biopsies, obtained before *E. coli* infection, vitamin D had no impact on tight junction proteins. However, during *E. coli* infection, vitamin D induced occludin and claudin-14 in mature superficial umbrella cells of the urinary bladder, as demonstrated by immunohistochemistry. Increased cell-cell adhesion consolidating the epithelial integrity is thereby promoted. We here describe a novel role of vitamin D in the urinary tract supporting vitamin D supplementation to restore the bladder epithelial integrity.

## Background

Recurrent urinary tract infections (UTI) are a major problem especially in postmenopausal women. This has partly been associated with low estrogen levels with an accompanying decrease of antimicrobial peptides and barrier proteins resulting in thinning of the urothelium and enhanced infection risk (Luthje et al. 2013). It is well-known that the permeability of the bladder urothelium increases, when tight junction proteins decrease allowing bacterial entry as well as passage of ions across the blood-urine barrier. To prevent bacterial invasion by forming a strong barrier, adjacent epithelial cells use the transmembrane proteins occludin, claudins and junctional adhesion molecules-1 (JAM-1). Not much is known about vitamin D in the context of tight junction proteins in the urinary tract. However, uropathogenic *Escherichia coli* (UPEC) infection disrupts the tight junction barrier with downregulation of occludin and claudins in bladder epithelial cells (Tian et al. 2018). Similarly, during interstitial cystitis syndrome, occludin is downregulated (Lee and Lee 2014). Avoiding infections by strengthening the urothelial barrier is therefore tempting. We here report the importance of sufficient vitamin D levels to ensure enough high levels of the barrier proteins occludin and claudin-14 in the urinary bladder of postmenopausal women and in mice during *E. coli* infection.

## Materials and methods

### Bacteria

Uropathogenic *E. coli* strain CFT073, expressing type 1, P and S fimbriae along with α-hemolysin, isolated from a patient with acute pyelonephritis was used in infection experiments. Bacteria were grown on a blood agar plate at 37°C overnight followed by 4 h in LB broth to obtain logarithmic phase of growth.

### Study participants

Postmenopausal women were supplemented with 2000 units of vitamin D_3_ (Recip, Meda Pharmaceuticals) daily for 12 weeks and analyses of serum 25D_3_ confirmed increased levels. None of them had any history of UTI and no one received any dietary supplements or hormonal treatment at the time of the study. Before vitamin D supplementation was initiated and after a 12-week follow-up, superficial biopsies were taken from the urinary bladder (Hertting et al. 2010).

### In vivo mouse model of UTI

Female wild-type C57BL/6 mice were obtained from Janvier Laboratories following the standard procedures. Mice were fed with normal diet. Similarly for vitamin D depleted C57BL/6 mice, 3-week-old animals were supplied with vitamin D-deficient diet TD.89123 for 7 weeks and were housed behind a UV protection film (Clear 1 UV, Data Solution, Sweden) directly after weaning, whereas control mice were fed with normal diet TD.110133 supplemented with 1.5 IU/g of cholecalciferol (Harlan Laboratories) (Hertting et al. 2017). Mice were anesthetized using isoflurane and transurethrally infected with 0.5 × 10^8^ colony-forming units of *E. coli* CFT073 in 50 μl of PBS. After 24 h infection, mice were sacrificed and their bladders were aseptically removed and fixed with 4% paraformaldehyde and processed for immunohistochemistry staining.

### Ex vivo infection of bladder biopsy

Bladder biopsies obtained from patients were immediately transferred to serum-free DMEM (Invitrogen) containing a low dose of gentamicin (1 μg/mL) with or without *E. coli* CFT073 at 10^8^ CFU/ml and incubated at 37 °C for 120 mins. Biopsies were then gently washed in PBS and fixed in 4% paraformaldehyde (PFA). Fixed tissue was embedded in paraffin, sectioned at 4 μm and processed for immunohistochemistry.

### Immunofluorescence staining of bladder sections

Sections of paraffin-embedded tissue were deparaffinized and rehydrated and pretreated with 0.3% Triton X-100/PBS at room temperature. Thereafter, sections were blocked for 30 mins with FX Signal Enhancer (Invitrogen); sections were blocked for an additional 60 mins with the sera from the species in which the secondary antibodies were raised. Incubation with primary antibodies was carried out overnight at 4 °C. Primary antibodies used were goat anti-claudin-14 (1:200; Abcam)and mouse anti-occludin (1:200; Santa Cruz Biotechnology). Sections were then incubated with secondary Alexa Fluor-conjugated antibodies (1:600; Invitrogen) for 60 mins at room temperature and mounted in ProLong Gold Antifade mounting medium including DAPI (Invitrogen). Tissue was analyzed with a Leica SP5 confocal microscope and quantified with ImageJ software.

### Statistical analysis

All statistical tests were performed in GraphPad Prism version 5. Data were obtained from Student’s unpaired or paired t-test as appropriate. Differences with *p* values below 0.05 were considered statistically significant.

## Results

**Fig. 1:**
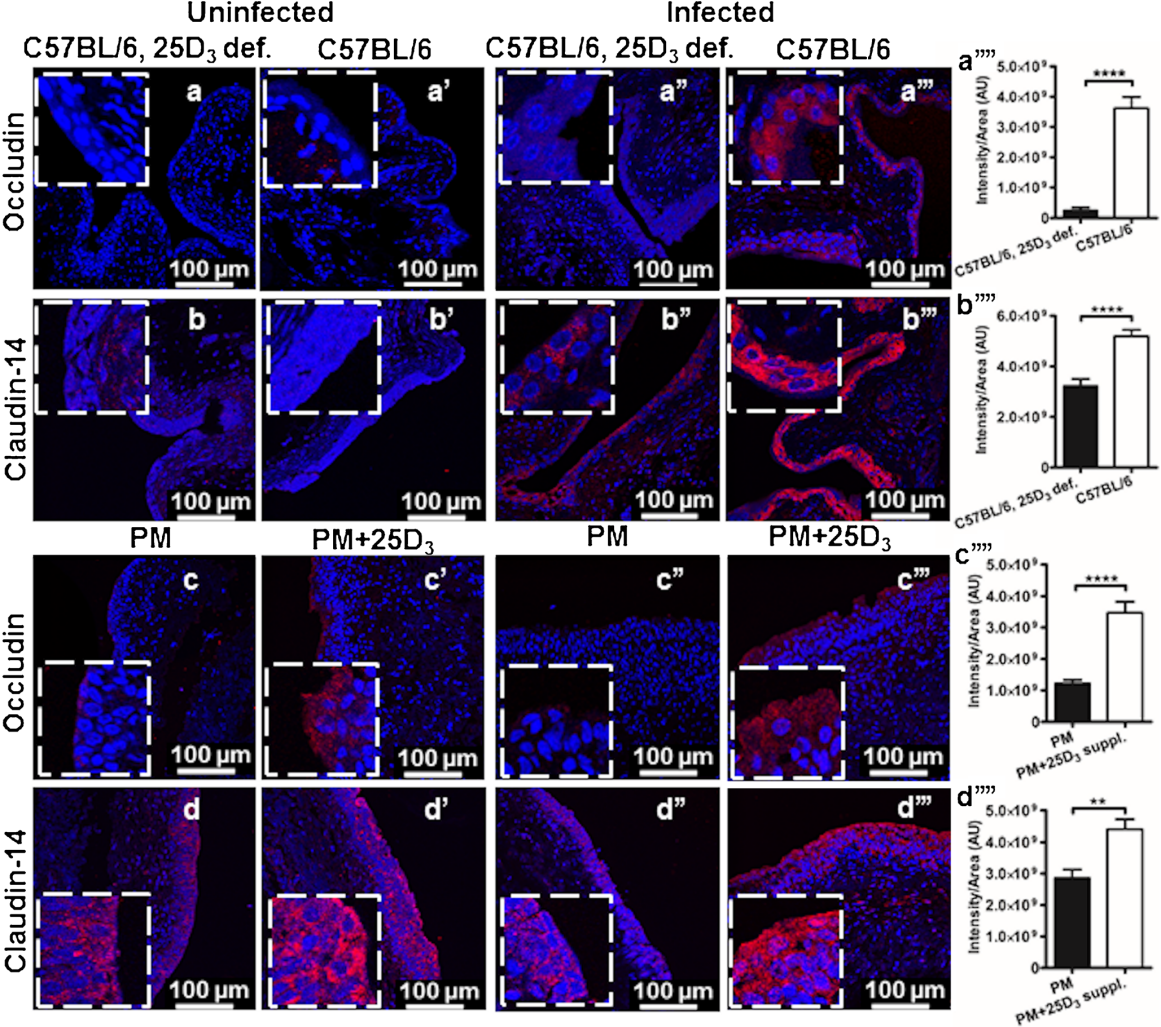
*E. coli* infectioninduced significant upregulation of tight junction proteins in vitamin D sufficient mice and human urinary bladders. Expression of the tight junction proteins occludin and claudin-14 in the mouse urinary bladder during vitamin D deficiency (*n* = 6) and sufficiency (*n* = 6) (**a, a’, b,b’**) without and (**a”, a”’, b”,b”’**) with transurethrally induced *E. coli* urinary tract infection. Bladder biopsies were obtained from postmenopausal women (*n* = 7) with insufficient vitamin D (25D_3_) levels before and after 12 weeks vitamin D supplementation. Biopsies were either (**c, c’, d,** and **d’**) uninfected or (**c”, c”’, d”,d”’**) *E. coli* infected ex vivo*.* Immunohistochemistry revealed no significant difference in (**a, a’, b,b’**) uninfected mice and (**c, c’, d,d’**) human bladders with or without sufficient vitamin D. Contrary, a significant upregulation as measured with densitometry of occludin and claudin-14 was observed in the urinary bladders of (**a””, b””**) *E. coli* infected vitamin D sufficient mice and (**c”’, d””**) ex vivo *E. coli* infected human bladder biopsies. Occludin and claudin-14 were localized in the superficial uroepithelium including the larger umbrella cells, lining the bladder lumen. Representative tissue samples are shown. Average mean fluorescence intensity per unit area is depicted in the right panel from three random view fields of each section. Images were captured at 40 × magnification and occludin and claudin-14 were stained with AlexaFluor 594 (red) and the cell nucleus with DAPI (blue). Zoomed images are shown as inserts, at 4 × further magnification. Data are shown as mean + SEM, **P < 0.01, ****P < 0.0001

In UTI, exfoliation of infected superficial bladder epithelial cells is an efficient strategy to shed invading pathogens. However, the loss of the superficial cells also facilitates bacterial invasion of underlying less-differentiated cells and establishment of persistent reservoirs. In this context, a strong epithelial barrier is important to prevent bacterial invasion and spread of infection. We sought to investigate if barrier cells can be tightened by vitamin D. This would imply a greater resistance to bacterial infection. To explore the effect, a vitamin D supplemented urinary bladder was analyzed without and with *E. coli* infection and the histological localization of occludin and claudin-14 was visualized using immunohistochemistry. In an uninfected mouse urinary bladder, vitamin D depletion did not influence the expression of occludin (Fig.1a, a') and claudin-14 (Fig. 1b, b’). However, *E. coli* infection in vitamin D supplemented wild-type mice had significantly higher expression levels of occludin (Fig. 1a”’) and claudin-14 (Fig. 1b”’) primarily localized in the superficial upper uroepithelial layers as compared to deficient-infected mice (Fig. 1a, b”). Densitometric analysis also confirmed the increased fluorescence intensity in the upper layers of the epithelium of *E. coli* infected vitamin D sufficient mice (Fig. 1a”, b,)”.

This observation prompted us to explore and confirm the effect of vitamin D in human urinary bladder. Bladder biopsies were obtained before and after supplementation and infected with uropathogenic *E. coli* CFT073 ex vivo. In line with our findings in mouse urinary bladder, vitamin D supplemented uninfected human urinary bladder biopsies did not impact the expression of occludin (Fig. 1c, c’) and claudin-14 (Fig. 1d, d’) when compared to control postmenopausal bladder biopsies. In contrast, *E. coli* infection significantly increased the expression of occludin (Fig. 1c”, c”’) and claudin-14 (Fig. 1d,” d”’) in the bladder biopsies after vitamin D supplementation, confirmed with densitometric analysis (Fig. 1c”,d”). Infection with *E. coli* CFT073 led to an increased expression of both occludin and claudin-14 predominantly in the upper layers of mature umbrella cells close to lumen, suggesting a local vitamin D response that further diminished towards the basal layers of lamina propria or submucosa.

## Discussion

Urothelial barrier function largely depends on the presence of tight junction proteins that not only regulate the paracellular permeability but also forms a strong barrier against invading pathogens. The tight junction proteins are present in the intersection of apical and lateral membranes of urothelial cells and help in the attachment of adjacent epithelial cells.

We here for the first time demonstrate that vitamin D induce tight junction proteins in the urinary bladder during *E. coli* infection. Our findings are in line with the involvement of vitamin D on tight junction proteins in the blood-brain barrier, where vitamin D deficiency decreased the expression of occludin and claudin-5 and is suggested to complicate stroke severity and chronic consequences in rat (Sayeed et al. 2019). Moreover, the vitamin D receptor was demonstrated to mediate the protective effect of vitamin D-induced expression of occludin, claudin-5 and zonula occludens in ischemic stroke (Won et al. 2015).

We showed the expression of occludin and claudin-14 in mature umbrella cells of the urinary bladder in both humans and mice. Previous studies showed occludin and different claudin family proteins localized in specific locations of the bladder tissue. In mouse, rat and rabbit bladders, occludin, claudin-8 and claudin-12 are specifically localized to the apicolateral tight junctions of the umbrella cell layer, whereas claudin-4 is also associated with the basolateral surface of the umbrella cells and the plasma membrane of the underlying cell layers (Acharya et al. 2004; Khandelwal et al. 2009). Similarly, in human ureters, claudin-3 is localized at the umbrella cell tight junction, whereas claudin-5 is expressed at the basolateral surface of the umbrella cell layer (Varley et al. 2006). Therefore, it is interesting that we detected occludin and claudin-14 in the superficial epithelial bladder cells with decreased expression in the basal layers in the urinary bladder, both in mice and postmenopausal women.

Impaired barrier function in junctional complexes facilitates translocation of luminal microbes into the epithelium. *S. aureus* has been shown to actively penetrate airway epithelia and secrete toxin protein A (SpA) resulting in the contraction of the cytoskeleton, causing a barrier defect (Soong et al. 2011). Similarly, uropathogenic *E. coli* infection promotes a severe urothelial barrier defect by promoting a paracellular permeability defect associated with the failure of umbrella cell tight junction zonula occludens 1 formation and umbrella cell sloughing (Wood et al. 2012). Likewise, *E. coli* lipopolysaccharides induced lung injury in mice, whereas vitamin D treatment preserved the alveolar barrier function by restoring to the normal level of occludin and zonula occludens 1 (Shi et al. 2016). Interestingly, vitamin D supplementation of initially vitamin D deficient mice had a limited effect on occludin and claudin-1 in lung epithelial cells, although it suppressed lung inflammation with a reduced IgM level and B cell activation in airway draining lymph nodes via increased expression of the vitamin D receptor (Gorman et al. 2017).

Vitamin D supplementation has been shown to induce antimicrobial peptides in the urinary bladder, keratinocytes and intestinal cells (Hertting et al. 2010; Huang 2016; Wang et al. 2004). Our current observations further add to the understanding of the multiple mechanisms by which vitamin D can act. They support the beneficial effect of supplementation to restore the epithelial integrity of the urinary bladder in postmenopausal women, thereby preventing bacterial invasion and recurrent UTI.

## Conclusion

Our data suggest that vitamin D upregulates tight junction proteins during *E. coli* infection, likely improving the epithelial integrity, which in turn may ameliorate the protection against infection. This finding is relevant especially among patients with recurrent UTI and where low vitamin D levels are anticipated.
